# Anti-obesity activity, acute toxicity, and chemical constituents of aqueous and ethanol *Viola mandshurica* extracts

**DOI:** 10.1186/s12906-017-1810-4

**Published:** 2017-06-06

**Authors:** Yoon-Young Sung, Dong-Seon Kim, Seung-Hyung Kim, Ho Kyoung Kim

**Affiliations:** 1 Mibyeong Research Center, Korea Institute of Oriental Medicine, 1672 Yuseong-daero, Yuseong-gu, Daejeon, 305-811 Republic of Korea; 2 KM Convergence Research Division, Korea Institute of Oriental Medicine, Daejeon, 305-811 Republic of Korea; 3 Institute of Traditional Medicine and Bioscience, Daejeon University, Daejeon, 300-716 Republic of Korea

**Keywords:** Adiponectin, Adipose tissue, AMP-activated protein kinase, Liver, Toxicity

## Abstract

**Background:**

*Viola mandshurica* has traditionally been used as an expectorant, diuretic, and anti-inflammatory drug. The present study was designed to test the hypothesis that low doses of two different *V. mandshurica* extracts have anti-obesity effects.

**Methods:**

We evaluated the effects of ethanol extract (VME) and aqueous extract (VMA) from *V. mandshurica* on high-fat diet (HFD)-induced obese mice as well as the acute oral toxicities and chemical compositions of both extracts.

**Results:**

Oral administration of VME or VMA (50, 100, or 200 mg/kg) decreased body weight gain, liver and adipose tissue mass, adipocyte size, and serum lipid levels. Both extracts increased adiponectin serum concentrations and mRNA expression in epididymal adipose tissue. VME and VMA also reversed the HFD-induced mRNA expression of lipogenic genes such as CCAAT/enhancer binding protein (C/EBP)α, C/EBPβ, sterol regulatory element-binding protein 1c, and leptin in adipose tissue, whereas they increased mRNA expression of uncoupling protein 2 and adenosine monophosphate-activated protein kinase (AMPK). VME and VMA increased the phosphorylation of AMPK and acetyl-coA carboxylase with a concomitant decrease in fat accumulation in the liver. High performance liquid chromatography analysis revealed that both VME and VMA contained esculetin (0.566% for VME, 0.231% for VMA) and schaftoside (0.147% for VME, 0.126% for VMA). In a 2-week acute toxicity study, administration of a single oral dose of VME or VMA (5000 mg/kg) caused no signs of toxicity or mortality.

**Conclusions:**

These results suggest that both VM extracts exert anti-obesity effects in HFD-induced obese mice by suppressing lipogenesis and activating AMPK in the liver and adipose tissue. Our findings suggest that VM extracts could be a safe and effective treatment for obesity.

## Background

Obesity is a major public health problem in developed countries because it conveys an increased risk of chronic metabolic diseases such as type 2 diabetes and coronary heart disease [1]. Various approaches to regulating obesity and weight have been suggested, including agents that inhibit nutrient absorption or appetite and induce weight loss [2]. Anti-obesity agents including orlistat, lorcaserin, phentermine/topiramate, and naltrexone/bupropion are currently approved for chronic weight management in obese adults [3], but they have considerable side effects such as nausea, vomiting, insomnia, constipation, headache, stomachache, and gastrointestinal disturbance [4, 5]. For these reasons, herbal medicines for weight control and obesity treatment have been used in many countries. Among them, extract obtained from *Garcinia cambogia* fruits containing hydroxycitric acid (HCA) has been routinely used for many centuries. Unlike chemical stimulants, it does not have toxic effects and is becoming a popular weight loss supplement [6, 7].

*Viola mandshurica* W. Becker is a perennial herb in the family *Violaceae* that is widely distributed in China, Korea, and Japan. It has traditionally been used because of its various pharmacological activities including diuretic, expectorant, and anti-inflammatory effects in conditions such as bronchitis, eczema, and skin eruptions [8]. *Viola* herbal extract is regarded to effectively clean blood components [9], and various studies show that *V. mandshurica* extract has potential anti-diabetic, anti-asthmatic, anti-oxidant, and neuroprotective activities [8, 10]. We previously showed that oral administration of *V. mandshurica* ethanolic extract (VME, 400 mg/kg/day) suppresses body weight and lipid accumulation in adipose tissue in mice [11]. These results were the first evidence that VME can ameliorate obesity in vivo. Here, we performed further studies on the dose-dependency, toxicity, and chemical characterization of VME and *V. mandshurica* aqueous extract (VMA). We also tested the effects of lower doses (50, 100, or 200 mg/kg) of VME and VMA on fat accumulation in the liver and adipose tissue of high-fat diet (HFD)-induced obese mice.

## Methods

### Plant material extraction

*V. mandshurica* was obtained as a dried whole plant from Omniherb Co. (Yeoungcheon, Korea) and authenticated using macroscopic and microscopic methods by the Classification and Identification Committee of the Korea Institute of Oriental Medicine. Voucher specimens (no. PH-88E, PH-88 W) were deposited at the herbarium of the Mibyeong research center at the Korea Institute of Oriental Medicine. *V. mandshurica* (100 g) was extracted twice with 70% ethanol or water using a 2-h reflux extraction, and the extract was concentrated under reduced pressure. The concentrate was filtered, lyophilized, and subsequently stored at 4 °C. The yields of the dried extract from starting crude materials were 11% and 19% (*w*/w) in 70% ethanol and water, respectively.

### High performance liquid chromatography (HPLC) analysis of VME and VMA

The standard compounds esculetin (6,7-dihydroxycoumarin, purity 98%) and schaftoside (6-C-beta-D-Glucosyl-8-C-alpha-L-arabinosylapigenin, purity 98%) were obtained from Sigma-Aldrich (St. Louis, MO, USA). HPLC-grade reagents, acetonitrile, and water were obtained from J. T. Baker (Phillipsburg, NJ, USA). All other chemicals used in this study were of reagent grade.

The chemical compositions of VME and VMA samples were analyzed by reverse phase-HPLC using a Waters Alliance 2695 system (Waters Co., Milford, MA, USA) coupled with a 2996 photodiode array detector. INNO C18 column (250 mm × 4.6 mm; particle size 5 μm, Phenomenex (Young JIN Biochrom Co., Ltd., CA, Korea)) was used as stationary phase, and the mobile phase was composed of 0.1% (*v*/v) trifluoroacetic acid/water (A) and acetonitrile (B). The gradient elution was optimized as shown in Table 1. The flow rate was 1.0 mL/min, and the column temperature was 40 °C. The injection volume was 20 μL.

**Table 1:** Composition of mobile phase with gradient elution

Time (Min)	A% (0.1% Trifluoroacetic Acid in Water)	B% (Actonitrile)
0–10	90	10
10	90	10
50	50	50
52	0	100
58	0	100
70	90	10
70	90	10

Identification was based on retention time and UV spectra by comparison with commercial standards. Components were quantified based on peak areas at maximum wavelengths for each compound. Quantification was performed using a mixture of external standards of known concentration that were analyzed in duplicate before and after the batch of samples, and peak areas were used to calculate the sample contents of the compounds. A calibration curve of the standards ranging from 0.5 to 4.0 mg/mL (four levels) revealed good linearity, with R^2^ values exceeding 0.99 (peak areas vs. concentration).

### Animals and diets

Seven-week-old male C57BL/6 J mice were purchased from Central Lab Animal Inc. (Seoul, Korea). Mice were housed in ventilated cages in an air-conditioned room at a temperature of 21 ± 2 °C and humidity of 50 ± 5% under a 12:12-h light/dark cycle. Mice were provided a commercial diet and water ad libitum for 1 week. All animal experiments were conducted in accordance with National Institutes of Health guidelines and approved by the Committee on Animal Care of the Daejeon University (Permit No. DJUARB2014–042). HFD mice were fed a diet (Rodent diet D12492, Research Diets, Inc., New Brunswick, NJ, USA) consisting of 60% fat, 20% protein, and 20% carbohydrate [12]. Normal control mice were fed a commercial standard diet (AIN-76A, Research Diets, Inc.) consisting of 11.5% fat, 20.8% protein, and 67.7% carbohydrate.

*Garcinia cambogia* extract and orlistat (Orlistat, Roche, Mannheim, Germany) were used as positive controls. Mice were randomly divided into ten groups (*n* = 12 per group): (1) normal diet with vehicle (ND-normal), (2) HFD with vehicle (HFD-control), (3) HFD with VME 200 mg/kg (HFD-VME), (4) HFD with VME 100 mg/kg, (5) HFD with VME 50 mg/kg, (6) HFD with VMA 200 mg/kg (HFD-VMA), (7) HFD with VMA 100 mg/kg, (8) HFD with VMA 50 mg/kg, (9) HFD with Orlistat (HFD-OR), and (10) HFD with *Garcinia cambogia* extract containing HCA (HFD-HCA). VME, VMA, OR, and HCA were dissolved in normal saline and administered orally every day for 7 weeks. OR and HCA were given at 50 and 100 mg/kg/day, respectively. The OR dose for mice was calculated based on the human dose. Body weight and food intake were measured once per week. Food efficiency ratio (FER, %) was calculated by the following formula: (body weight gain [g/day]/food intake [g/day]) × 100.

### Tissues weight and histological analysis

At the end of the experimental period, mice were fasted for 15 h and then sacrificed. After blood collection, subcutaneous, epididymal, and kidney white adipose tissue and the liver, kidney, and spleen were removed and immediately weighed. For adipocyte staining, epididymal adipose tissue and the liver were fixed in 10% neutral formalin solution for 24 h and then embedded in paraffin. All tissue was cut to a thickness of 6 μm and stained with hematoxylin and eosin (H&E) or Oil Red O. Adipocyte size was determined from stained tissue sections under light microscopy (Olympus BX51, Olympus Optical Co., Tokyo, Japan). Immunohistochemistry was performed to evaluate AMPK and acetyl-coA carboxylase (ACC) activation in the liver. Phosphorylation of both proteins was detected in immersion-fixed paraffin-embedded sections of the liver using a 1:100 dilution of p-AMPKα rabbit antibody (Cell Signaling, Danvers, MA, USA) or p-ACC rabbit antibody (Cell Signaling) at 4 °C overnight.

### Serum biochemical parameter analysis

Blood samples were centrifuged at 2000 *g* for 20 min at 4 °C, and the serum was stored at −70 °C until analysis. Serum levels of triglyceride, free fatty acid, glucose, total cholesterol, high-density lipoprotein (HDL)-cholesterol, low-density lipoprotein (LDL)- cholesterol, alanine aminotransferase (ALT), aspartate aminotransferase (AST), and creatinine were analyzed with an automatic analyzer (Hitachi-720, Hitachi Medical, Japan). Serum leptin and adiponectin concentrations were measured with immunoassays using commercially available kits (Linco Research, St. Charles, MO, USA).

### Real-time reverse-transcriptase polymerase chain reaction (PCR)

Total RNA from epididymal adipose tissue was isolated using a homogenizer and TRI reagent (Sigma-Aldrich). Total RNA (5 μg) was used to synthesize cDNA with the First Strand cDNA Synthesis kit (Amersham Pharmacia, Little Chalfont, UK). Real-time quantitative PCR was performed using the Applied Biosystems 7500 Real-Time PCR system (Applied Biosystems, Foster City, CA, USA). The probes were labeled with the fluorescence reporter dye 6-carboxy-fluorescein (FAM, Applied Biosystems). The endogenous control was the mouse glyceraldehyde-3-phosphate dehydrogenase (GAPDH) probe (VIC/MGB probe, primer limited, 4352339E, Applied Biosystems). PCR conditions were 2 min at 50 °C, 10 min at 94 °C, 15 s at 95 °C, and 1 min at 45 °C for 40 cycles. The expression levels of mRNA were normalized to those of GAPDH and calculated using the 2^-△△Ct^ method according to the manufacturer’s instructions. Specific primer sequences for target genes are shown in Table 2.

**Table 2:** Primer sequences

Genes	Probe and Primer	Sequence	GenBank Accession Number
Leptin	Forward	5'-CCAAAACCCTCATCAAGACC-3'	NM_008493
	Reverse	5'-GTCCAACTGTTGAAGAATGTCCC-3'	
UCP2	Forward	5'-CCGCATTGGCCTCTACGACTCT-3'	NM_011671
	Reverse	5'-CCCCGAAGGCAGAAGTGAAGTG-3'	
Adiponectin	Forward	5'-CCCAAGGGAACTTGTGCAGGTTGGATG-3'	NM_009605.4
	Reverse	5'-GTTGGTATCATGGTAGAGAAGAAAGCC -3'	
C/EBPα	Forward	5'-TGGACAAGAACAGCAACGAGTAC-3'	NM_001287523.1
	Reverse	5'-CGGTCATTGTCACTGGTCAACT-3'	
C/EBPβ	Forward	5'-AAGCTGAGCGACGAGTACAAGA-3'	NM_001287739.1
	Reverse	5'-GTCAGCTCCAGCACCTTGTG-3'	
SREBP1c	Forward	5'-AGCCTGGCCATCTGTGAGAA-3'	XM_006532714.2
	Reverse	5'-CAGACTGGTACGGGCCACAA-3'	
AMPK α1	Forward	5'-AAGCCGACCCAATGACATCA-3'	XM_011245321.1
	Reverse	5'-CTTCCTTCGTACACGCAAAT-3'	
AMPK α2	Forward	5'-GATGATGAGGTGGTGGA-3'	NM_178143.2
	Reverse	5'-GCCGAGGACAAAGTGC-3'	
GAPDH	VIC	5'-TGCATCCTGCACCACCAACTGCTTAG-3'	

### Acute toxicity test

To meet guidelines for toxicity testing (2014–136) from Korea’s Ministry of Food and Drug Safety (MFDS) at BIOTOXTECH, a Good Laboratory Practice institute, a single 5000 mg/kg dose was given to ten Sprague Dawley rats (five males and females, obtained from Orient Bio Inc., Seoul, Korea). Control rats were treated with vehicle. Animals were individually observed for clinical signs of toxicity, body weight change, and mortality at 30, 60, 120, 240, and 360 min after dosing; periodically during the first 24 h; and daily thereafter for a total of 14 days.

### Statistical analysis

All data are presented as mean ± standard error of the mean (SEM), and differences were considered statistically significant at *p* < 0.05. Differences between groups were examined using unpaired Student’s *t*-tests and analyses of variance (ANOVA) followed by Duncan’s multiple range tests.

## Results

### Effects of VME and VMA on body weight, FER, and organ weights

**Fig. 1:**
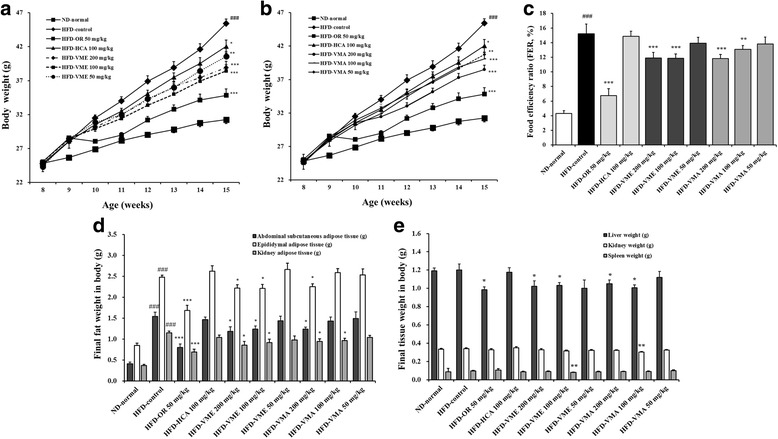
Effects of VME and VMA on HFD-induced obese mice. **a**-**b** Body weight, **c** FER, **d** fat weight, and **e** liver, kidney, and spleen tissue weights. Data are presented as mean ± SEM (*n* = 12); #*p* < 0.05, ##*p* < 0.01, ###*p* < 0.001 vs. ND-normal; *p < 0.05, **p < 0.01, ***p < 0.001 vs. HFD-control

Figure 1 shows body weight (g), FER (%), and final organ weight (g) during the 7-week experimental period. After 7 weeks, the HFD group had a higher mean body weight than the ND group, but oral administration of VME or VMA reduced body weight in HFD mice (Fig. 1a-b) without changing food intake (data not shown). The mean FER was significantly higher in the HFD group than in the ND group, but oral administration of VME or VMA at doses of 100 and 200 mg/kg reduced FER (Fig. 1c). The weights of abdominal subcutaneous, epididymal, and kidney adipose tissue were significantly increased in the HFD group, but VME or VMA at 200 mg/kg decreased these weights (Fig. 1d). In particular, VME at a dose of 100 mg/kg inhibited the increased weight of all types of adipose tissue. Compared with the HFD group, significant decreases in liver weight were observed in the VME and VMA groups (100 and 200 mg/kg) and the OR group (Fig. 1e). VME and VMA (100 mg/kg) slightly reduced spleen and kidney weights, respectively (Fig. 1e). Thus, VME and VMA may decrease body and organ weight without influencing food intake. Neither VME nor VMA induced any adverse effects.

### Effects of VME and VMA on serum levels of lipids, tissue function indicators, and adipokines

**Fig. 2:**
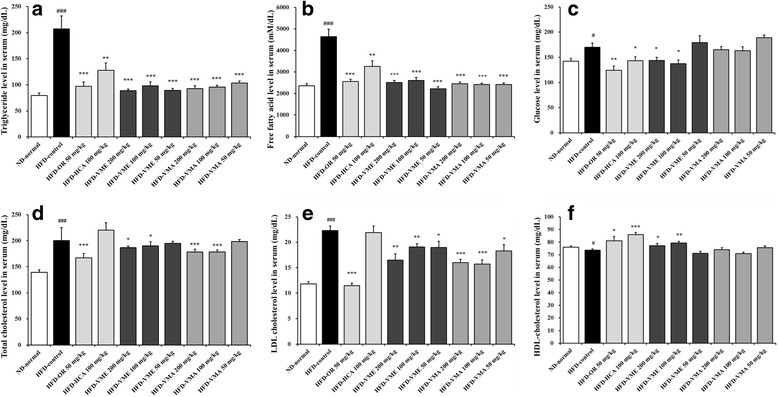
Effects of VME and VMA on serum lipid levels in HFD-induced obese mice. **a** Triglyceride, **b** free fatty acid, **c** glucose, **d** total cholesterol, **e** LDL-cholesterol, and **f** HDL-cholesterol levels. Data are presented as mean ± SEM (*n* = 12); #*p* < 0.05, ##*p* < 0.01, ###*p* < 0.001 vs. ND-normal; *p < 0.05, **p < 0.01, ***p < 0.001 vs. HFD-control

Serum triglyceride, free fatty acid, total cholesterol, and LDL-cholesterol levels in the HFD group were significantly higher than those in the ND group, but oral administration of VME and VMA decreased these levels (Fig. 2a-b and d-e). Orlistat had similar effects, but total cholesterol and LDL-cholesterol levels did not change in the HCA group. The glucose level in the HFD group was significantly higher than that in the ND group but was decreased by OR, HCA, and VME (100 and 200 mg/kg) (Fig. 2c). HDL-cholesterol levels were increased in the HFD-OR, HFD-HCA, HFD-VME 100 mg/kg, and HFD-VME 200 mg/kg groups (Fig. 2f).

**Fig. 3:**
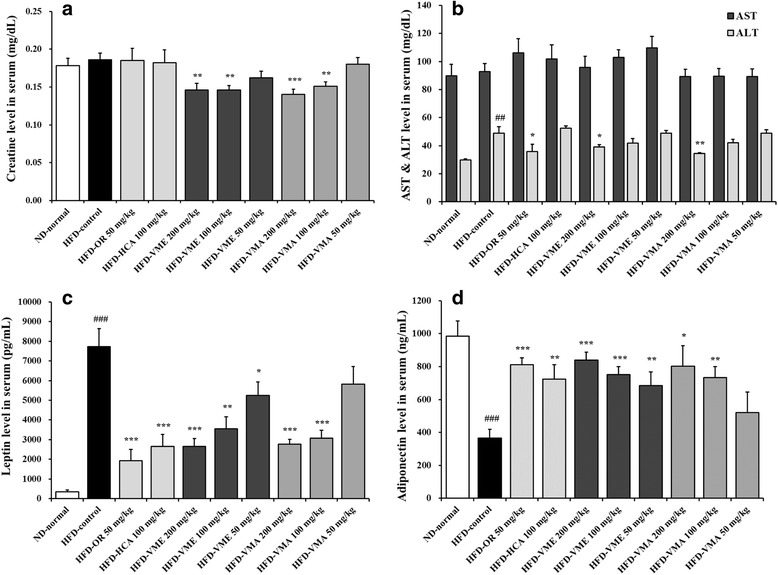
Effects of VME and VMA on serum biochemical parameters in HFD-induced obese mice. **a** Creatinine, **b** AST and ALT, **c** leptin, and **d** adiponectin levels. Data are presented as mean ± SEM (*n* = 6–12); #*p* < 0.05, ##*p* < 0.01, ###*p* < 0.001 vs. ND-normal group; *p < 0.05, **p < 0.01, ***p < 0.001 vs. HFD-control

The serum concentrations of creatinine, AST, and ALT were measured as indicators of renal and liver function. Serum creatinine concentrations in the HFD-VME and HFD-VMA groups were significantly decreased relative to the HFD-control group (Fig. 3a). Serum AST levels did not differ among groups. ALT level in the HFD-control group was significantly increased, but VME and VMA (200 mg/kg) inhibited the increase in ALT. These results indicate that oral administration of VME or VMA induced no detectable adverse toxic effects in mice.

The mean serum leptin level was significantly higher in the HFD-control group than in the NC-normal group, but leptin levels in VME- and VMA-treated groups were significantly decreased (Fig. 3c). The serum adiponectin level in the HFD group was lower than that in the NC group, but adiponectin level was significantly increased in the VME (50, 100, and 200 mg/kg) and VMA (100 and 200 mg/kg) groups as well as the OR and HCA groups (Fig. 3d).

### Effects of VME and VMA on adipocyte size

**Fig. 4:**
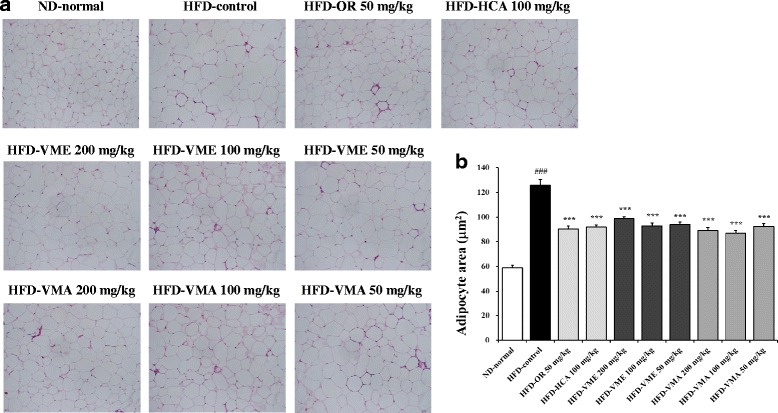
Effects of VME and VMA on histology of adipose tissue in HFD-induced obese mice. **a** Epididymal adipose tissue morphology and **b** adipocyte area. Representative images of H&E-stained sections. Data are presented as mean ± SEM (*n* = 4); #*p* < 0.05, ##*p* < 0.01, ###*p* < 0.001 vs. ND-normal; *p < 0.05, **p < 0.01, ***p < 0.001 vs. HFD-control

Histological analysis showed larger adipocyte size in epididymal adipose tissue from the HFD-control group compared with that from the ND-normal group. VME and VMA effectively inhibited the increase in adipocyte size, as did OR and HCA (Fig. 4).

### Effects of VME and VMA on mRNA expression levels of lipid metabolism-related genes in epididymal adipose tissue

**Fig. 5:**
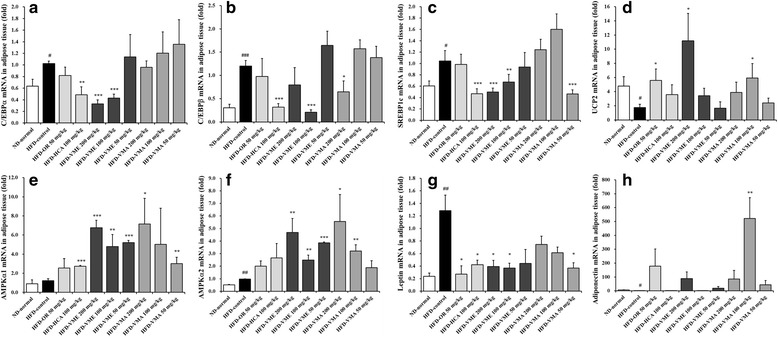
Effects of VME and VMA on mRNA levels in epididymal adipose tissue. **a** C/EBPα, **b** C/EBPβ, **c** SREBP1c, **d** UCP2, **e** AMPKα1, **f** AMPKα2, **g** leptin, and **h** adiponectin. Data are presented as mean ± SEM (*n* = 4); #*p* < 0.05, ##*p* < 0.01, ###*p* < 0.001 vs. ND-normal; *p < 0.05, **p < 0.01, ***p < 0.001 vs. HFD-control

We next analyzed the mRNA levels of genes involved in adipogenesis in adipose tissue after 7 weeks of VME or VMA administration (Fig. 5). mRNA levels of the adipogenic-related transcription factors CCAAT/enhancer binding protein (CEBP)α, CEBPβ, and sterol regulatory element-binding protein (SREBP)1c were significantly increased in the HFD group but decreased in the VME and VMA groups compared with those in the ND group (Fig. 5a-c). Thermogenesis-related mitochondrial uncoupling protein (UCP)-2 mRNA expression was significantly decreased in the HFD group and was markedly higher in the VME and VMA groups (Fig. 5d). AMPKα1 and AMPKα2 mRNA levels were significantly increased in the VME and VMA groups (Fig. 5e-f). Moreover, VME and VMA decreased and increased leptin and adiponectin mRNA expression, respectively (Fig. 5g-h).

### Effects of VME and VMA on liver histology and AMPK expression

**Fig. 6:**
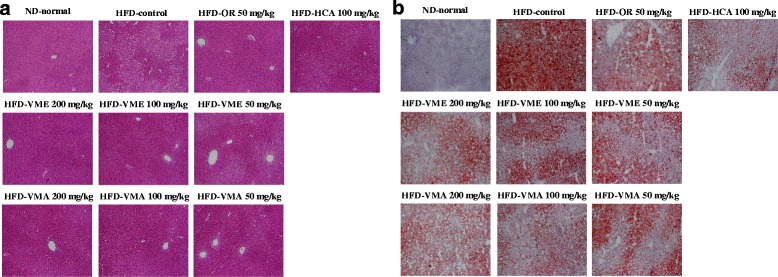
Effects of VME and VMA on liver morphology in HFD-induced obese mice. Tissue sections stained with **a** H&E and **b** Oil Red O

The effects of VME and VMA on liver morphology and lipid accumulation were determined by H&E and Oil Red O staining, respectively. The HFD-control group had larger lipid droplets and more excess lipid accumulation than the ND-normal group, but lipid droplet size and lipid accumulation were decreased in VME and VMA groups (Fig. 6).

**Fig. 7:**
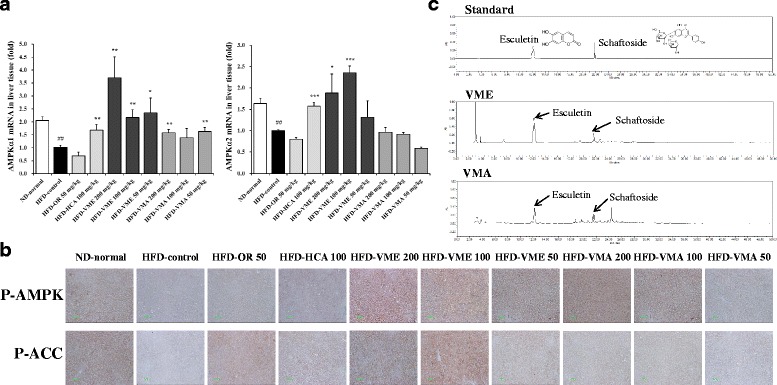
Effects of VME and VMA on hepatic AMPK expression and HPLC chromatogram. **a** AMPKα1 and AMPKα2 mRNA expression and **b** AMPK and ACC phosphorylation. **c** HPLC-PDA chromatograms of two standards mixture, VME, and VMA at 330 nm. Esculetin and schaftoside appeared at retention times of approximately 12.3 and 21.7 min, respectively. Data are presented as mean ± SEM (*n* = 4); #*p* < 0.05, ##*p* < 0.01, ###*p* < 0.001 vs. ND-normal; *p < 0.05, **p < 0.01, ***p < 0.001 vs. HFD-control

AMPK plays a key role in energy homeostasis and cellular glucose and lipid metabolism [13]. Thus, we investigated whether VME and VMA affect AMPK mRNA expression and activation in the liver. AMPKα1 and AMPKα2 mRNA levels were decreased in the HFD group but were increased in the HFD-VME and HFD-VMA groups (Fig. 7a). VMA only increased the expression of AMPKα1 mRNA. Next, we examined the phosphorylation of AMPK and its downstream target enzyme, ACC (Fig. 7b). The administration of VME and VMA effectively increased the phosphorylation of AMPK and ACC compared with the HFD group, but this effect was stronger for VME.

### Chemical composition of VME and VMA

Quantitative HPLC analysis was performed for standardization of VM extracts. HPLC analysis showed that VME contained 5.66 ± 0.07 mg/g esculetin and 1.47 ± 0.05 mg/g schaftoside, whereas VMA contained 2.31 ± 0.03 mg/g esculetin and 1.26 ± 0.02 mg/g schaftoside (Fig. 7c).

### Acute toxicity test

An acute toxicity test revealed that oral administration of a single 5000 mg/kg dose of VME or VMA induced no signs of toxicity, body weight change, or mortality in rats during a 14-day observation period.

## Discussion

We investigated the anti-obesity and lipid-lowering effects of two *V. mandshurica* extracts in HFD-induced obese mice. After 7 weeks, VME and VMA decreased body weight gain, FER, fat and liver masses, serum lipid levels, and adipocyte size relative to HFD mice. The expression of lipid metabolism-related genes and AMPK, an important energy regulator, were significantly altered in VME- and VMA-treated HFD mice. By contrast, HCA, a positive control compound extracted from *Garcinia cambogia* fruit, significantly decreased body weight and serum triglyceride but did not dramatically affect the food efficiency ratio, fat mass, or serum total cholesterol and LDL-cholesterol levels. These results suggest that both VM extracts have potent anti-obesity effects.

To elucidate the mechanisms by which VME and VMA exert anti-obesity effects, we investigated lipid metabolism and AMPK signaling in the liver and adipose tissue. Activation of AMPK in both types of tissue plays a major role in regulating glucose and lipid metabolism via the stimulation of fatty acid oxidation and inhibition of lipogenesis and glucose production [14, 15]. Given the role of AMPK signaling pathway-related proteins in glucose and lipid metabolism, it is important to identify and analyze their mRNA and protein expression levels in adipose tissue and the liver. Thus, we investigated whether VME and VMA affect mRNA expression and activation of AMPK and AMPK signaling pathway-related-proteins in these tissues. Gene expression analyses showed that VME and VMA decreased the mRNA levels of lipogenic genes such as C/EBPα, C/EBPβ, SREBP1c, and leptin in the epididymal adipose tissue of HFD-induced obese mice. VME and VMA also increased mRNA levels of the thermogenesis-related protein UCP2 [16] along with AMPKα1 and AMPKα2 in adipose tissue. In addition, VME and VMA markedly increased adiponectin serum levels and mRNA expression in adipose tissue. The effects of fat cell-derived adiponectin on insulin-sensitizing and fatty-acid-oxidizing actions is dependent on AMPK activation (i.e., phosphorylation) in the adipose tissue and liver [17, 18]. We previously reported that VME induces AMPK activation and inactivation of ACC, a downstream target of AMPK, in adipose tissue [11]. In the present study, we demonstrated that mRNA and phosphorylation of AMPKα decreased in the liver of obese mice, suggesting that alterations in AMPKα expression contribute to the pathogenesis of lipid accumulation in the liver of obese mice. However, VME and VMA stimulated AMPK expression and activation and inhibited ACC activation in the liver, suggesting that VME and VMA improve abnormal lipid metabolism by suppressing lipogenesis and promoting fatty acid oxidation via up-regulation of AMPK.

HPLC analysis revealed that both VME and VMA contain esculetin and schaftoside. Esculetin reportedly inhibits adipogenesis in 3 T3-L1 adipocytes [11, 19]. Esculetin at a concentration of 10 μg/mL inhibits adipocyte differentiation to 31% of fully differentiated cells, whereas VME at the same concentration has no significant effect [11]. Oral administration of esculetin (30 mg/kg) to HFD-induced obese mice decreases body weight and reduces serum levels of total triglyceride, total cholesterol, and glucose [20]. The free hydroxyl groups in the chemical structures of these coumarin compounds are favorable for anti-adipogenesis activity and lipolysis [20]. Recently, the c-glycosylflavone compound schaftoside was reported to exhibit pancreatic lipase inhibitory activity [21]. We found that the amount of esculetin in VME was 2.14-fold greater than that in VMA, which might be one reason for the greater suppressive effect of VME on fat accumulation in adipose tissue. These results suggest that esculetin and schaftoside are partly responsible for the anti-obesity effects of VM extracts. However, we did not determine whether the effects of VMA and VME are dependent on these two compounds because we administered a complex mixture to the mice. Previous phytochemical studies show that Herba Violae mainly contains several flavonoids, coumarins, and organic acids [22]. Thus, further studies are needed to determine the anti-obesity effects of particular components through the phytochemical analysis of bioactive compounds in VM extracts.

Oral administration of a single 5000 mg/kg dose of VME or VMA caused no signs of toxicity or mortality in ten rats. Thus, the LD_50_ values of VME and VMA are likely higher. Compounds with an LD_50_ over 2000 mg/kg are considered relatively safe according to the Globally Harmonized System of Classification and Labelling of Chemicals [23] and acute toxicity guidelines of the Organization for Economic Cooperation and Development [24]. Hence, these results suggest that both VM extracts are reasonably safe for traditional medical use.

## Conclusions

Oral VME and VMA reduced body weight gain, fat mass, and serum lipid concentrations in HFD-induced obese mice. A single 5000 mg/kg dose of these extracts appeared to have no adverse effects in rats. Overall, our findings suggest that VME and VMA exert anti-obesity effects in HFD-induced obese mice by activating AMPK and suppressing lipid metabolism in adipose tissue and the liver. Therefore, VME and VMA should be further investigated as anti-obesity and anti-hyperlipidemia agents.

## Abbreviations

ACC: acetyl-coA carboxylase
ALT: alanine aminotransferase
AMPK: adenosine monophosphate-activated protein kinase
AST: aspartate aminotransferase
C/EBP: CCAAT/enhancer binding protein
GAPDH: glyceraldehyde-3-phosphate dehydrogenase
HCA: hydrocitric acid
HDL: high-density lipoprotein
HFD: high-fat diet
LD: lethal dose
LDL: low-density lipoprotein
ND: normal diet
OR: orlistat
SREBP1c: sterol regulatory element-binding protein1c
UCP: uncoupling protein
VMA: *Viola manshurica* aqueous extract
VME: *Viola mandshurica* ethanolic extract
